# Primary care physicians’ own exercise habits influence exercise counseling for patients with chronic kidney disease: a cross-sectional study

**DOI:** 10.1186/1471-2369-15-48

**Published:** 2014-03-19

**Authors:** Yoshiyuki Morishita, Akihiko Numata, Atushi Miki, Mari Okada, Kenichi Ishibashi, Fumi Takemoto, Yasuhiro Ando, Shigeaki Muto, Daisuke Nagata, Eiji Kusano

**Affiliations:** 1 Division of Nephrology, Department of Medicine, Jichi Medical University, 3311-1, Yakushiji, Shimotsuke-city, Tochigi 329-0498, Japan; 2 Department of Medical Physiology, Meiji Pharmaceutical University, Kiyose, Japan; 3 Department of Internal medicine, Ustunomiya Social Insurance Hospital, Ustunomiya, Japan

## Abstract

**Background:**

The appropriate exercise counseling for chronic kidney disease (CKD) patients is crucial to improve their prognosis. There have been few studies about exercise counseling by primary care physicians for CKD patients. We investigated primary care physicians’ exercise counseling practices for CKD patients, and the association of these physicians’ own exercise habits with exercise counseling.

**Methods:**

The population of this cross-sectional study was 3310 medical doctors who graduated from Jichi Medical University from 1978 to 2012. The study instrument was a self-administered questionnaire that was mailed in August 2012 to investigate their age class, specialty, workplace, exercise habits, and practices of exercise counseling for CKD.

**Results:**

581 (64.8%) medical doctors practiced the management of CKD among a total of 933 responses. These 581 medical doctors were defined as CKD primary care physicians and their answers were analyzed. CKD primary care physicians’ own exercise habits (frequencies and intensities) were as follows: frequencies: daily, 71 (12.1%); ≥2–3 times/week, 154 (26.5%); ≥1 time/week, 146 (25.1%); and ≤1 time/month, 176 (30.2%); intensities: high (≥6 Mets), 175 (30.1%); moderate (4–6 Mets), 132 (22.7%); mild (3–4 Mets), 188 (32.3%); very mild (<3 Mets), 47 (8.1%); and none, 37 (6.4%). The CKD primary care physicians’ exercise recommendation levels for CKD patients were as follows: high, 31 (5.3%); moderate, 176 (29.7%); low, 256 (44.0%); and none, 92 (15.8%). The CKD primary care physicians’ exercise recommendations for CKD patients were significantly related to their own exercise frequency (p < 0.001), but they were not related to their age, specialty, workplace, or exercise intensity.

**Conclusions:**

CKD primary care physicians’ exercise recommendation level for CKD patients was limited. In addition, CKD primary care physicians’ own exercise habits influenced the exercise counseling for CKD patients. The establishment of guidelines for exercise by CKD patients and their dissemination among primary care physicians are needed.

(University Hospital Medical Information Network Clinical Trial Registry. number, UMIN000011803. Registration date, Sep/19/2013)

## Background

Chronic kidney disease (CKD) patients show gradual decline in maximal exercise capacity in accordance with the progression of their CKD stage [1,2]. Actually, CKD stage 3–5 patients have been reported to show lower peak oxygen consumption, averaging 50-80% compared with healthy subjects [3,4]. This decreased exercise capacity decreases quality of life and enhances sarcopenia, which can be defined as the age-related (1% per year after the age of 25) loss of muscle [5,6]. Several studies reported that sarcopenia progressed much more intensively in CKD patients in association with several factors often observed in CKD, such as nutritional deficiencies, acidosis, and vitamin D deficiency [7,8]. Sarcopenia is an independent predictor of low physical performance activities and fractures [9]. Recently, accumulated evidence has demonstrated that all CKD patients, irrespective of CKD stage and treatment modality, can improve their physical functioning and reduce the risk of sarcopenia by exercise training [10,11]. Previous studies have reported beneficial effects of resistance exercise training on muscle mass and contractile function in CKD patients [12-15]. In addition, several studies also have reported the beneficial effects of exercise on potential mediators of cardiovascular disease in CKD [12,16]. Since the prevalence of CKD has been increasing globally [17,18] and it has become a common disease globally, like cardiovascular diseases and metabolic syndrome, appropriate exercise counseling by primary care physicians as well as nephrologists for CKD patients is crucial to improve their prognosis. However, physicians reported limited medical school and residency training education about the beneficial effects of exercise, as well as inadequate guidelines for writing exercise prescriptions or referrals as barriers to exercise counseling [19,20]. Several studies reported that physicians who exercise are more likely to counsel their patients to do so as well [21,22]. However, there have been few studies about exercise counseling by primary care physicians for CKD patients. Therefore, in the present study, we investigated primary care physicians’ exercise counseling practices for CKD patients. Furthermore, we also investigated the associations of primary care physicians’ own exercise habits with their exercise counseling, as well as major barriers for exercise counseling for CKD patients.

## Methods

This study was conducted in accordance with the Declaration of Helsinki and was approved by the ethics committee of Jichi Medical University. Since this study was analysis of anonymous self-administered questionnaire, responses were considered as consents for this study. This study was registered at University Hospital Medical Information Network Clinical Trial Registry (UMIN-CTR). The identification number is UMIN000011803.

### Subjects

The population of this cross-sectional study was 3310 medical doctors who graduated from Jichi Medical University from 1978 to 2012. The medical doctors who graduate from this medical university have a 5–7-year term of duty to work in a rural area of Japan as primary care physicians. In addition, almost all medical doctors (> 80%) continue to work as primary care physicians after this term of duty.

### Study instrument

The study instrument was a self-administered questionnaire. This was designed to obtain detailed information about primary care physicians’ characteristics, including their age class, specialty, workplace, personal exercise habits, and management of CKD (exercise counseling practice, medical prescription pattern). The results of their medical prescription pattern will be analyzed and reported elsewhere. This study focused on the primary care physicians’ exercise counseling practices for CKD patients, and the association of primary care physicians’ own exercise habits with their exercise counseling for CKD. The questions that were used for analysis in the present study were as follows:

Characteristics

1. Age (24–30, 30–40, 40–50, 50–60, ≥60 years)

2. Specialty (internal medicine, surgery, general medicine, pediatrics, others)

3. Workplace: (university hospital, polyclinic hospital, hospital, clinic, others)

Personal exercise habits

1. Exercise frequency (daily, ≥2–3 times/week, ≥1 time/week , ≤1 time/month)

2. Exercise intensity (high [≥6 Mets], eg, swimming, jogging, soccer, cycling; moderate [4–6 Mets], eg, quick walking, golf; mild [3–4 Mets], eg, walking, cleaning; very mild [<3 Mets], eg, stretching, cooking; none; others)

Exercise recommendation for CKD

1. Management of CKD patients (yes, no)

2. Diseases for which you recommend exercise [multiple selection] (diabetes mellitus, hyperlipidemia, apoplexia, heart failure, arterial hypertension, others)

3. General exercise recommendations for CKD patients (high, moderate, low, others)

The next two questions were for those who chose high or moderate for the question above about general exercise recommendations for CKD patients.

1. Recommended exercise frequency for CKD patients (daily, ≥2–3 times/week, ≥1 time/week, ≤1 time/month)

2. Recommended exercise intensity for CKD patients(high [≥6 Mets], eg, swimming, jogging, soccer, cycling; moderate [4–6 Mets], eg, quick walking, golf; mild [3–4 Mets],eg, walking, cleaning, very mild [<3 Mets],eg, stretching, cooking; others

The next question was for those who chose low or no for the above question about general exercise recommendations for CKD patients.

1. What are the barriers for exercise recommendations for CKD patients [multiple selection] (no interest, inadequate knowledge on the effects of exercise, concern that exercise may impair renal function and cause complications, inadequate knowledge to prescribe exercise for CKD patients, inadequate time, others)

### Statistical analysis

The associations between primary care physicians’ own exercise habits (frequency and intensity) and their age class, specialty, and workplace were analyzed by multiple linear regression analysis to determine the independent variables. Values of p < 0.01 were considered to be significant.

## Results

The survey was mailed to 3310 medical doctors, with responses being received from 933 in total (28.2%). 37 responses were excluded from this study due to their inadequacy. Among the remaining 896, 581 (64.8%) medical doctors managed CKD patients. In this study, these 581 medical doctors were defined as CKD primary care physicians and their answers to the self-administered questionnaire were analyzed.

### The characteristics and exercise habits of CKD primary care physicians

The characteristics of CKD primary care physicians’ age class, specialty, workplace and their exercise habits are shown in Table 1 and Table 2. Multivariable linear regression analysis showed that the CKD primary care physicians’ exercise frequency was significantly associated with their age class (p < 0.01), but was not associated with their specialty or workplace (Table 3). Those in the older age classes were more likely to have a high exercise frequency. The CKD primary care physicians’ exercise intensity was not significantly associated with their age class, their specialty and workplace (Table 3).

**Table 1:** The characteristic of CKD primary care physicians

**Age**	**Number (%)**	**Specialty**	**Number (%)**	**Workplace**	**Number (%)**
24-30	55 (9.5)	Internal medicine	350 (60.2)	University hospital	51 (8.8)
30-40	189 (32.5)	Surgery	48 (8.3)	Polyclinic hospital	89 (15.1)
40-50	175 (30.1)	General medicine	145 (25.0)	Hospital	187 (32.1)
40-50	154 (26.5)	Pediatrics	12 (2.1)	Clinic	239 (41.1)
60≦	8 (1.4)	Others	26 (4.5)	Others	15 (2.6)
**Total**	**581 (100)**	**Total**	**581 (100)**	**Total**	**581 (100)**

*Abbreviations*: *CKD* chronic kidney disease.

**Table 2:** CKD primary care physicians’ exercise habits

**Frequency**	**Number (%)**	**Intensity**	**Number (%)**
Daily	71 (12.2)	High (≥6 Mets)	175 (30.1)
≥2–3 times/week	154 (26.5)	Moderate (4–6 Mets)	132 (22.7)
≥1 time/week	146 (25.1)	Mild (3–4 Mets)	188 (32.2)
≤1 time/month	176 (30.3)	Very mild (<3 Mets)	47 (8.1)
Others	32 (5.5)	None	37 (6.4)
N/A	2 (0.3)	Others	3 (0.5)
**Total**	**581 (100)**	**Total**	**203 (100)**

*Abbreviations*: *CKD* chronic kidney disease, *N/A* not available.

**Table 3:** Multivariate linear regression analyses of the association of age, specialty and workplace and exercise habits (frequency and intensity) in CKD primary care physician

	**Exercise frequency**		**Exercise intensity**	
	**(Model R**^**2**^ **= 0.139)**		**(Model R**^**2**^ **= 0.139)**	
	**χ**^**2**^	**p**	**χ**^**2**^	**p**
Age (years old)	38.224	*0.001	37.462	0.010
Specialty	28.470	0.028	25.553	0.181
Workplace	18.623	0.289	21.999	0.341

*p < 0.01.

### CKD primary care physicians’ exercise counseling

**Figure 1:**
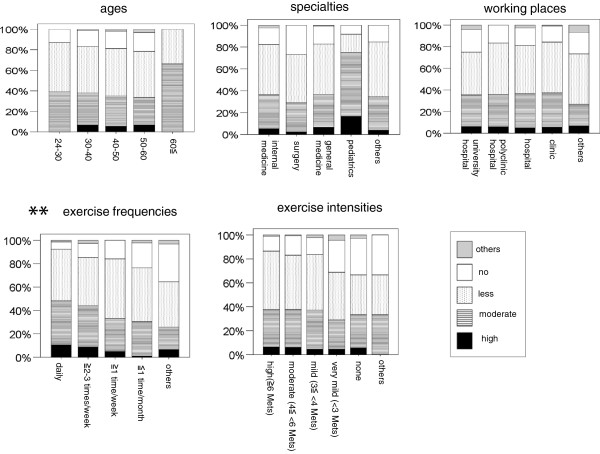
**The associations of CKD primary care physicians’ exercise recommendations (general) for CKD patients and their age class, specialty, and workplace, as well as their own exercise frequency and intensity**. CKD: chronic kidney disease. **p < 0.001.

**Figure 2:**
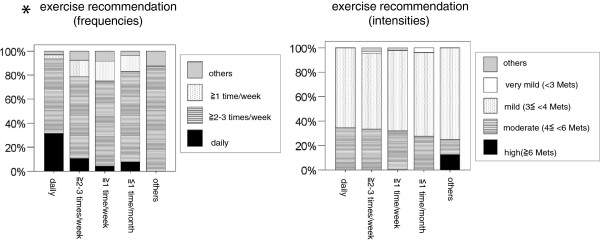
**The associations of CKD primary care physicians’ own exercise frequency and their exercise recommendations (frequency and intensity) for CKD patients.** CKD: chronic kidney disease. *p < 0.01.

The diseases for which CKD primary care physicians recommend exercise were as follows: diabetes mellitus, 562 (96.7%); hyperlipidemia, 509 (87.6%); apoplexia, 250 (43.0%); heart failure, 129 (22.2%); arterial hypertension, 408 (70.2%); and others, 61 (10.5%). Table 4 shows CKD primary care physicians’ exercise counseling for CKD patients. Among CKD primary care physicians who had low and no exercise recommendations, the barriers to exercise counseling for CKD patients are shown in Table 5. Multivariable linear regression analysis showed that the CKD primary care physicians’ exercise recommendations (general) for CKD patients were significantly associated with their own exercise frequency (p < 0.001), but they were not associated with their age, specialty, workplace, or their own exercise intensity (Table 6, Figure 1). The CKD primary care physicians who had a high exercise frequency were more likely to recommend exercise at a high frequency for CKD patients than those who had a lower exercise frequency (Figure 1). Furthermore, multivariable linear regression analysis showed among the CKD primary care physicians who had high and moderate exercise recommendations for CKD patients, exercise recommendations frequency was associated with their own exercise frequency (p < 0.01) but not exercise intensity (Table 7, Figure 2). They were not also associated with their age class, specialty or workplace (Table 7). The CKD primary care physicians who had a high exercise frequency were more likely to recommend exercise at a high frequency for CKD patients (Figure 2). Their exercise recommendations intensity for CKD patients was not associated with their age class, specialty, or workplace, or with their own exercise frequency and intensity (Table 6).

**Table 4:** CKD primary care physicians’ exercise counseling for CKD patient

**General**	**Number (%)**	**Frequency**	**Number (%)**	**Intensity**	**Number (%)**
High	31 (5.3)	daily	22 (10.8)	High (≥6 Mets)	1 (0.5)
Moderate	172 (29.6)	≥2–3 times/week	143 (70.4)	Moderate (4–6 Mets)	61 (30.5)
Low	256 (44.1)	≥1 time/week	25 (12.3)	Mild (3–4 Mets)	132 (66.0)
No	92 (15.8)	≤1 time/month	0 (0)	Very mild (<3 Mets)	3 (1.5)
Others	10 (1.7)	Others	13 (6.4)	None	3 (1.5)
N/A	20 (3.4)			Others	3 (1.5)
				N/A	3 (1.5)
Total	581 (100)	Total	203 (100)	Total	203 (100)

*Abbreviations*: *CKD* chronic kidney disease, *N/A* not available.

**Table 5:** Barriers for CKD primary care physicians’ exercise counseling for CKD patient

**Barrier**	**Number (%)**
No interest	11 (3.2)
Inadequate knowledge on the effects of exercise	204 (58.6)
Concern that exercise may impair renal function and cause complications	113 (32.5)
Inadequate knowledge to prescribe exercise for CKD patients	234 (67.2)
Inadequate time	21 (6.0)
Others	21 (6.0)

*Abbreviation*: *CKD* chronic kidney disease.

**Table 6:** Multivariate linear regression analyses of the association of age, specialty and workplace and exercise habits of CKD primary care physician and their exercise counseling (general) for CKD patient

	**Exercise recommendation general**	
	**(Model R**^**2**^ **= 0.333)**	
	**χ**^**2**^	**p**
Age (years old)	20.616	0.194
Specialty	19.744	0.232
Workplace	11.937	0.748
Exercise frequency	38.548	** < 0.001
Exercise intensity	13.055	0.875

*Abbreviation*: *CKD* chronic kidney disease, **p < 0.001.

**Table 7:** Multivariate linear regression analyses of the association of age, specialty and workplace and exercise habits of CKD primary care physician who had who had high and moderate exercise recommendations for CKD patients and their exercise counseling (frequency and intensity) for CKD patient

	**Exercise recommendation frequency**		**Exercise recommendation intensity**	
	**(Model R**^**2**^ **= 0.333)**		**(Model R**^**2**^ **= 0.333)**	
	**χ**^**2**^	**p**	**χ**^**2**^	**p**
Age (years old)	9.137	0.691	16.973	0.387
Specialty	19.619	0.075	17.649	0.345
Workplace	19.378	0.080	12.986	0.674
Exercise frequency	28.728	*0.004	16.693	0.406
Exercise intensity	17.719	0.278	26.126	0.162

*Abbreviation*: *CKD* chronic kidney disease; *, p < 0.01.

## Discussion

The results in the present study show that primary care physicians’ exercise recommendation levels for CKD patients were limited because their rate of positive exercise recommendations (high recommendation: 5.4% + moderate recommendation: 30.9%) was < 40% and their rate of negative exercise recommendations was > 50% (low recommendation: 44.1% + no recommendation: 15.8%) for such patients. In addition, the CKD primary care physicians’ exercise recommendation (general) for CKD patients was significantly associated with their own exercise frequencies, but not with their age, specialty, workplace or their own exercise intensity. Furthermore, in the CKD primary care physicians who had positive exercise recommendations (high and moderate) for CKD patients, their exercise frequency recommendations for CKD patients were significantly associated with their own exercise frequency; however, they were not associated with their age class, specialty, or workplace, or with their own exercise intensity, and their exercise intensity recommendations for CKD patients were not associated with their age class, specialty, or workplace, or with their own exercise frequency and intensity.

There have been few studies about exercise counseling by primary care physicians for CKD patients. To the best of our knowledge, this is the first study to report on such exercise counseling. Several studies reported that physicians who have substantial exercise habits are more likely to counsel their patients to exercise [21,22]. In the present study, we also found associations of the CKD primary care physicians’ exercise frequency recommendations with their own exercise frequency.

Recently, several studies have demonstrated that all CKD patients, irrespective of their CKD stage, treatment modality, age, and functional impairment, can benefit from exercise [10-15]. Exercise including resistance exercise training can improve their physical capacity and reduce the risk of sarcopenia [10-15]. In addition, although the direct cardiovascular outcome in CKD patients due to exercise has not been reported, several studies have reported the beneficial effects of exercise on potential mediators of cardiovascular disease such as arterial stiffness, C-reactive protein, and interleukin 6 [12,16]. In terms of renal function, although there have been no large studies that clearly showed the effects of exercise on renal function, several studies have shown that exercise decreased proteinuria and glomerular sclerosis in an animal model with CKD [23,24]. The European Association of Rehabilitation in Chronic Kidney Disease recommends maintaining CKD patients on a fairly intense level of exercise [25]. The National Kidney Foundation Kidney Disease Outcome Quality Initiative (K/DOQI) clinical practice guidelines recommend that physical functioning assessment and encouragement to participate in physical activity should be part of the routine care plan for dialysis patients [26]. However, these recommendations do not seem to have been widely adopted and have been insufficiently referred to CKD primary care physicians because positive exercise recommendations by CKD primary care physicians in the present study were limited at <40% (high recommendation: 5.4% + moderate recommendation: 30.9%), and the rate of negative exercise recommendation was > 50% (low recommendation: 44.1% + no recommendation: 15.8%) for CKD patients; however, they highly recommended exercise for patients with metabolic syndrome, such as diabetic mellitus (97.0%), hyperlipidemia (87.9%), and hypertension (70.8%). In addition, in the CKD primary care physicians who had negative exercise recommendations for CKD patients, the main reasons why these physicians did not recommend exercise for CKD patients in the present study were inadequate knowledge on the effects of exercise (59.3%) and inadequate knowledge to prescribe exercise (68.7%). Each CKD patient has a different type of disease, such as cardiovascular disease, heart disease, and metabolic syndrome. These complicated conditions may contribute to difficulty in establishing clear exercise guidelines for CKD patients. Clyne reported that physicians should preferably prescribe exercise training and nephrologists or physiologists should design a program and evaluate its progress for CKD patients [25]. The cooperative planning and management of exercise for CKD patients may be useful to encourage exercise and improve their prognosis. In the management of CKD patients, appropriate pharmacological medication in CKD also very impotent. We investigated and reported medication-prescribing patterns of the primary care physicians in CKD. In that analysis, there were certain associations between the prescribing patterns of the CKD primary care physicians for CKD and their specialty and workplace (in press); however, there was no association between their prescribing patterns for CKD and their exercise habits (unpublished data).

There are several limitations in this study. First, this was cross-sectional study, which limits the generalizability of the results. Seconds, since the study instrument was a mailed self-administered questionnaire, there may be self-selection bias, and it may also contribute to low response rate (28.2%) to questionnaire. Second, the primary care physicians in this study may not be a representative population of all primary care physicians because all medical doctors in this study graduated from one medical university; however, majority of graduates of this medical university work as primary care physicians. Third, the results were from a self-administered questionnaire and were not objectively evaluated in terms of primary care physicians’ personal exercise habits and exercise recommendations for CKD patients. These confounding factors in cross-sectional study might affect the results. Further studies will thus need to investigate more accurately the exercise prescription patterns for CKD patients and the exercise habits of primary care physicians using instruments such as exercise recording devices for both primary care physicians and CKD patients.

## Conclusion

CKD primary care physicians’ exercise recommendation level for CKD patients was limited. In addition, CKD primary care physicians’ own exercise habits influenced the exercise counseling for CKD patients. The establishment of guidelines for exercise by CKD patients and their dissemination among primary care physicians are needed.

## Competing interests

The authors declare no competing interests.

## Authors’ contributions

YM participated in the design of the study and coordination, and performed the statistical analysis and drafted the manuscript. AN participated in its design. AM participated in its design and helped to carry out the statistical analysis. MO participated in its design and helped to carry out the statistical analysis. KI participated in its design. FT participated in its design. YA participated in its design. SM participated in its design. DN conceived of the study and participated in its design and coordination. EK conceived of the study and participated in its design and coordination. All authors have read and approve the final manuscript.

## Pre-publication history

The pre-publication history for this paper can be accessed here:

http://www.biomedcentral.com/1471-2369/15/48/prepub
